# Sun exposure and health safety practices of high school students in an urban population of Iran

**DOI:** 10.1186/s12889-019-8100-7

**Published:** 2019-12-26

**Authors:** Hosna Janjani, Saharnaz Nedjat, MasoudYunesian, Simin Nasseri, Fatemeh Haghighat Doost, Ramin Nabizadeh

**Affiliations:** 1 Department of Environmental Health Engineering, School of Public Health, Tehran University of Medical Sciences, Tehran, Iran; 2 Department of Epidemiology and Biostatistics, School of Public Health, Tehran University of Medical Sciences, Tehran, Iran

**Keywords:** Sun protection, Health safety, Behavior, Skin cancer, Students

## Abstract

**Background:**

Skin cancer attributed to sunlight exposure has an increasing trend worldwide, resulting in increased problems for healthcare services. This study aimed to evaluate the health safety practices of high school students in Saveh, Iran according to a sun protection guideline produced locally for Iran.

**Method:**

This cross-sectional study was done in 31 government and private high schools of Saveh, a central city of Iran. A sun protection questionnaire was designed in accordance with Iran sun protection guideline which has been developed based on the World Health Organization and US Environmental Protection Agency guidelines. The questionnaire consists of four sections of demographic information, knowledge, attitude, and protection behavior. A total of 504 students were selected by simple random sampling method. Pearson correlation coefficient and multiple linear regression analysis were used to assess the correlation between knowledge, attitude and behavior of the participants.

**Results:**

The mean score of the students’ knowledge, attitude, and behavior regarding harmfulness of ultraviolet radiation was 50.79 ± 14.64, 60.41 ± 15.04, and 45 ± 11.59, respectively. A significant association was observed between attitude and knowledge, behavior and knowledge as well as attitude. There was a significant correlation between sex and protective behaviors, sex and knowledge (*p* ≤ 0.001), and education level and knowledge (*p* = 0.002).

**Conclusion:**

students who protected themselves from sunlight less than others believed that their protection level was adequate. Health concerns related to sun exposure suggest the need for increasing the student’s awareness in sun protection area. As behavior is influenced by cultural aspects, implementation of the current guideline can be effective in reducing the health effects of sun exposure.

## Background

Sunlight radiation, including UVA, UVB is known to cause cancer [1]. Based on the International Agency for Research on Cancer (IARC), the US National Toxicology Program (NTP), and the US Environmental Protection Agency (EPA), ultraviolet radiation is carcinogenic to humans. Major health problems linked to UV radiation exposure are categorized as skin cancer (melanoma and non-melanoma), premature aging and other skin damage, cataracts and other eye damage, and Immune system suppression [2]. Based on several studies, ultraviolet (UV) exposure is responsible for 90% of non-melanomas and 65% of melanomas of global skin cancers [3]. Skin cancer, as the main side effect of UV radiation, is the most commonly diagnosed cancer in many countries including Denmark, Australia, United States, and France, and is associated with significant mortality and morbidity [4].

Reports indicate a growing trend in skin cancer worldwide [5]. The American Cancer Society (ACS) reported the prevalence of Melanoma skin cancer in females in 2016 and showed a significant increasing trend in the survival rate over the past three decades (82, 88, and 93%, respectively) [6]. Incidence rate of skin cancer have increased in Iran, where one of the most prevalent type of cancer is skin cancer [7].

The increasing trend of skin cancer incidence is a challenge for healthcare services [7]. This trend has led researchers to study the awareness, attitude, and behavior of individuals regarding sunlight exposure in order to identify the predisposing factors of skin cancer and to perform improvement interventions. To the best of our knowledge little is known about the experience of sun protection behavior in the Iran population. Moreover, sun safety educational approaches are not implemented in Iran***.*** Assessing the people’s awareness and behavior regarding sunlight protection is very important in implementing training and prevention programs. Also, the measurement criteria and evaluation questionnaires are very important in designing interventions and providing a comprehensive evaluation. Accurate evaluation tools and assessing different aspects of the people’s information and details of avoidance behaviors provide a precise estimate of their knowledge and behavior. In this study, questionnaires were developed based on the current sun protection guidelines of Iran to assess the knowledge, attitude, and health safety behavior of high school students in Saveh, Iran.

## Methods

This cross-sectional study was conducted in Saveh, Iran and the reporting of this study is compliant with STROBE guidelines (see Additional file 2). The present study aimed to evaluate the health safety practices of high school students against sun radiation and to know the students’ attitude and knowledge about the risks of sun radiation based on the questionnaire developed according to a sun protection guideline produced locally for Iran. All government and private high schools (34 high schools) in Saveh with a population of 10,555 students (4734 males and 5821 females) were considered as the statistical population of this study. Three schools were excluded due to lack of cooperation and 31 schools considered in the present study. By setting the value of *P* = 0.5, d = 0.05 and α = 0.05 sample size was obtained 385 and taking into account the cluster sampling design effect, the sample size was considered 504.All 31 schools included in the present study and 4 students from each course of schools were selected through simple random sampling and considering the following inclusion criteria. Inclusion criteria were age between 14 to 19 years, not be under dermatological therapy and live in Saveh., Also, the participants were excluded if they did not agree to participate in the study. Questionnaires were distributed among the students and the objective of the study was explained to the students. Also, it was clearly described that our project was approved by Tehran University of Medical Sciences (N4180001) and confidentiality and consent was desirable from all the participants. Prior to the data collection, written informed consent obtained from the participants and they were informed that participation was voluntary. Then they were asked to complete the questionnaires if they were willing to. Demographic data were analyzed and the average score of knowledge, attitude, and behavior were calculated. Pearson correlation coefficient and multiple linear regression analysis were applied to assess the correlation between knowledge, attitude, and behavior of the participants. The SPSS software version 19 was used for data analysis. The following questionnaires were used in this study.

### Sun protection questionnaire

A sun protection questionnaire was developed in accordance with the Iran sun protection guideline. This guideline has been prepared for Iran based on the World Health Organization (WHO) and US Environmental Protection Agency guidelines. In other words, we wished to test the sun protection guideline in Iran. The questionnaire had four sections. The first section was related to demographic information (age, sex, students’ educational level, parents educational level and self-reported complexion considering Fitzpatrick skin prototype: Pale white, white, olive to brown, dark brown, black) [8]. Other sections were related to knowledge, attitude and protection behavior. The knowledge section contained 40 questions on a three-point Likert scale for correct answers [1], incorrect answers (0), and I do not know (0). The attitude section had 5 questions in a 5-point Likert scale (Strongly disagree = 0, disagree = 1, undecided = 2, agree = 3, strongly Agree = 4). The behavior section had 2 sub-scales: high-risk behaviors for tanning (5 questions in a 5-point Likert scale as never = 5, rarely = 4, sometimes 3, often = 2, always = 1) and protection measures (15 questions in a 5-point Likert as never = 1, rarely = 2, sometimes = 3, often = 4, always = 5). The questionnaire is provided in the Additional file 1.

After designing the questions related to each of the considered areas, the reliability, validity (including relevance, clarity, and content validity), Item Content Validity Index (I-CVI), Scale Content Validity Index (S-CVI), and general comprehensiveness of the tool were assessed. The steps were completed with the participation of 6 content specialists and 6 potential participants. To convert comments to a quantitative scale, the appropriateness and clarity of each question and the appropriateness and general clarity of the tool were divided into four categories as 1: unfavorable, 2: rather favorable, 3: desirable, and 4: perfectly favorable. Moreover, the general comprehensiveness of the tool was divided into four categories as 1: incomplete), 2: rather comprehensive, 3: comprehensive, and 4: completely comprehensive. After collecting the experts’ opinions, the appropriateness and clarity of each question and the appropriateness and clarity of the whole tool were evaluated and necessary changes were made. The appropriateness and general clarity of the revised tool were 82 and 84 according to experts and 89 and 91 according to participants (out of 100), respectively. Moreover, the general comprehensiveness of the tool was 100 according to experts.

### Reliability of the questionnaire

The reliability of the questionnaire was determined by means of Cronbach’s alpha coefficient to assess its internal consistency and test-retest in 30 students in two sessions 2 weeks apart to assess its repeatability. In this study, the Cronbach’s alpha of the questionnaire for knowledge, attitude, and behavior was 0.6, 0.72, and 0.8 respectively, representing the acceptable reliability of the tools. The ICC of knowledge, attitude, and behavior was 0.73, 0.85, and 0.84 respectively.

## Results

After calculating the scores of knowledge, attitude, and behavior, all scores were converted to a range of 0–100. In order to better assess the level of knowledge, attitude, and protective behaviors of the students, the scores obtained in these areas were divided into four categories as 0–25 (very poor), 25–50 (poor), 50–75 (moderate), and 75–100 (good). Based on the results of Table 1, the majority of the participants (69.8%) were selected from government schools. More than half of the participants were females (55.55%). The age range of the participants in this study was between 14 and 19 years and the majority of them had a white complexion (55.68%). The mean score of the students’ knowledge of the harmfulness of ultraviolet radiation and protection ways was 50.79 ± 14.64 (moderate). Moreover, the mean score of attitude and protective behaviors was 60.41 ± 15.04 (moderate) and 45 ± 11.59 (poor). The mean score of high-risk behaviors for tanning was 77.76 ± 14.7 in all students, 79.04 in males, and 73.93 in females. Evaluation of the frequency of sunburn during the last 12 months revealed that most of the students (66.47%, *n* = 335) did not experience sunburn, 25.79% (*n* = 130) experienced sunburn once every 6 months, 5.55% (*n* = 28) once every 2–3 months, 1.59% (*n* = 8) every month, and few students (0.59%, n = 3) experienced sunburn every week during last 12 months.

**Table 1:** Demographic characteristics of study participants

Demographic characteristics (*N* = 504)		Government school, n (%)	Private school n (%)	Total n (%)
Sex	Female	192 (54.55)	88 (57.89)	280 (55.55)
	Male	160 (45.45)	64 (42.11)	224 (44.44)
Age (year)	14	29 (8.24)	11 (7.24)	40 (7.93)
	15	82 (23.30)	40 (26.32)	122 (24.20)
	16	91 (25.85)	50 (32.89)	141 (27.98)
	17	100 (28.41)	28 (18.42)	128 (25.40)
	18	49 (13.92)	21 (13.82)	70 (13.89)
	19	1 (0.28)	2 (1.31)	3 (0.60)
Educational level	First year of high school	88 (25.00)	48 (31.58)	136 (28.98)
	Second year of high school	88 (25.00)	48 (31.58)	136 (28.98)
	Third year of high school	88 (25.00)	28 (18.42)	116 (23.02)
	Fourth grade of high school	88 (25.00)	28 (18.42)	116 (23.02)
Complexion	Pale white	15 (4.26)	4 (2.63)	19 (3.77)
	White	196 (55.68)	88 (57.90)	284 (56.35)
	Olive to brown	141 (40.06)	60 (39.47)	198 (39.29)

Based on Table 2, the females ‘mean score of knowledge and protective behavior was higher compared to males. A significant correlation was detected between the students’ education level and knowledge (*p* = 0.002). Moreover, there was a significant association between the student’s attitude and complexion (*P* = 0.021). The score of knowledge, attitude, and behavior of government school students were higher compared to private school students. The highest mean score of knowledge was seen in sophomores with a white complexion, whose fathers had at least a high school diploma and mothers had higher levels of education. Furthermore, the highest mean score of attitude and protective behaviors was seen respectively in seniors and juniors who had a pale white complexion.

**Table 2:** The Pearson correlation between knowledge, attitude and protective behaviors with demographic variables

Demographic characteristics		Knowledge		Attitude		Protective behaviors	
		Mean (SD)	*P* Value	Mean (SD)	*P* Value	Mean (SD)	*P* Value
Sex	Female	53.72 (15.29)	< 0.001	60.71 (15.28)	0.608	49.50 (11.25)	< 0.001
	Male	47.12 (12.9)		60.02 (14.75)		39.37 (9.30)	
Kind of school	Government	51.44 (14.4)	0.135	60.80 (15.5)	0.340	45.27 (11.79)	0.407
	Private	49.28 (15.1)		59.47 (13.91)		44.36 (11.07)	
Educational level	First year of high school	47.94 (14.31)	0.002	58.93 (15.53)	0.512	45.52 (11.68)	0.445
	Second year of high school	54.65 (16.53)		60.99 (15.34)		45.35 (12.35)	
	Third year in high school	50.04 (11.77)		60.21 (13.88)		45.53 (11.35)	
	Fourth grade of high school	50.34 (14.46)		61.63 (15.24)		43.43 (10.70)	
Father Educational level	Illiterate	47.81 (12.44)	0.586	61.56 (14.80)	0.984	46.87 (9.38)	0.706
	Elementary	50.58 (15.50)		61.05 (15.58)		46.02 (11.46)	
	Middle school	49.86 (14.20)		60.03 (13.90)		44.18 (11.91)	
	High school and diploma	52.02 (14.86)		60.18 (14.71)		45.28 (11.78)	
	Academic	50.14 (14.34)		60.58 (17.08)		44.22 (11.17)	
Mother Educational level	Illiterate	50.17 (10.16)	0.641	67.50 (13.69)	0.093	48.21 (7.55)	0.696
	Elementary	49.55 (15.33)		58.73 (14.72)		44.54 (11.17)	
	Middle school	51.51 (14.38)		59.63 (15.56)		44.89 (11.40)	
	High school and diploma	50.64 (14.38)		60.72 (14.10)		45.57 (11.62)	
	Academic	52.78 (15.09)		63.52 (16.76)		44.07 (13.49)	
Complexion	Pale white	49.47 (16.57)	0.671	69.73 (17.19)	0.021	47.96 (11.91)	0.427
	White	51.28 (14.62)		60.19 (14.84)		45.13 (11.61)	
	Olive to brown	50.21 (14.51)		59.82 (14.89)		44.53 (11.50)	

Based on Table 3, there was a weak positive correlation between attitude and sun protection knowledge. Furthermore, there was a moderate positive correlation between behavior and knowledge and a weak positive correlation between behavior and attitude.

**Table 3:** Pearson correlation between knowledge, attitude and behavior

	Knowledge (r)	Attitude (r)	Behavior (r)
Knowledge1			
Attitude	0.097	1	
Behavior	0.306	0.18	1

**Fig. 1:**
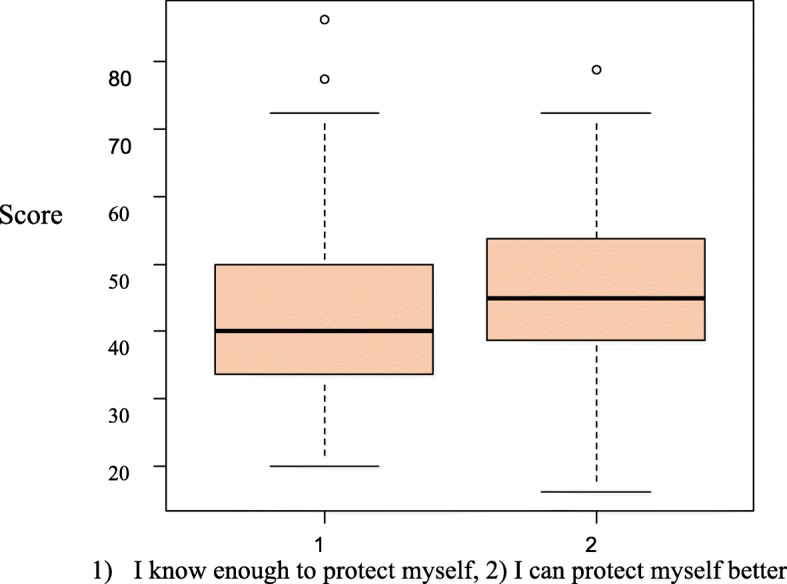
Students’ opinions about their protective behavior

Based on Fig. 1, the mean score of sun protection behaviors was lower in those who had more knowledge in this regard compared to the students who believed they had little information and needed to increase their protection level. In fact, students who protected themselves from sunlight less than others believed that their protection level was adequate.

**Fig. 2:**
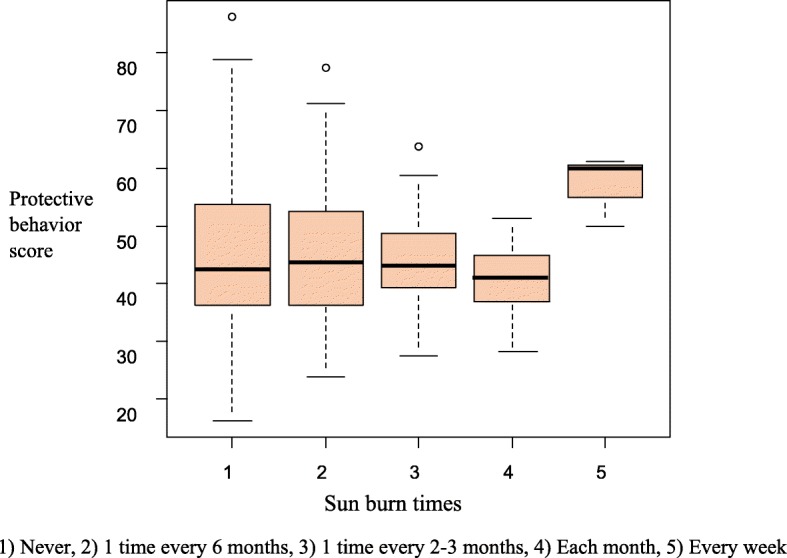
Students’ score of protective behaviors and frequency of sunburn

Based on Fig. 2, the mean score of protective behaviors was 45.08 ± 12.07 in students who never had sunburn, 44.93 ± 11 in students who had sunburn every 6 months, 44.28 ± 8.77 in students who had sunburn every 2–3 months, 40.62 ± 7.29 in students who had sunburn every month, and 57.08 ± 6.16 in students who had sunburn every week.

## Discussion

According to the results of the present study, the mean score of the students’ knowledge and attitude was moderate and the mean score of sun protection behaviors was poor. The age range of the study population had a significant share in the Iranian population age pyramid. Therefore, any type of intervention and education aimed at increasing the students’ awareness of the protection behaviors from sunlight can play an important role in reducing the risk of potentially harmful levels of sunlight exposure. It should be noted that this study considered all schools in Saveh and a large population included as a sample. Also the study considered the sun protection guideline for designing the questionnaire and the recommendation from this study can be considered in decision-making in this area. In a study conducted in fifth grade students from 10 schools throughout Framingham, Massachusetts, the results showed that sun protection programs in early childhood may not equally benefit everyone when applied to the general population. As children have certain molecular-level alterations predisposing them to increased complications of exposure to sunlight regardless of the level of UVB exposure, it is necessary to apply sun protection programs more seriously [9].

Evaluation of knowledge, attitude, and behavior of college students attending a large Midwestern university about melanoma and sun protection revealed a low level of knowledge of melanoma and sun prevention behaviors. Moreover, the students’ attitude toward sun protection was in the moderate range and their general sun protection behaviors and specific sunscreen use behaviors were fairly weak [10]. A similar study was carried out in the general population of Saudi Arabian. The results revealed a low-level knowledge towards sun exposure dangers and sun protection measures; moreover, sun protection was generally insufficient in adults [11]. The results of a study conducted in Turkey high schools showed that students did not have enough knowledge about and proper care against sunlight and needed training in this regard [12] and based on other study conducted in Turkey, students did not regularly practice sun protection behavior [13]. Other studies conducted in China, America, Spain, Morocco, etc. suggests a need to pay more attention to this issue [14–17].

In the present study, despite the relatively good attitude of students towards the impact of protective methods, these methods were not widely used. Based on our study results and other studies conducted in this area, it seems despite relatively good awareness of the youth about sun protection, they do not put their knowledge into practice [18, 19].

The results of the present study showed a significant correlation between sex and the students’ knowledge and protection behavior. Furthermore, the mean scores of knowledge, attitude, and behavior of females were higher compared to the scores of males. A study of high school students in Sakarya, Turkey about knowledge, behavior, and attitude toward sun protection showed that girls’ scores of knowledge and behavior were higher than boys’ scores, although boys had better attitude scores [20]. This finding is also consistent with the results of studies conducted in Miami and Connecticut [21, 22]. As females receive more counseling for beauty and makeup, they have more information about taking care of sunlight and protect themselves better. Furthermore, some cultural differences such as men not applying sunscreens may play an important role in reducing their scores of attitude and sun protection behavior. In a study conducted in Thailand, boys did not have adequate protection against sunlight such as using sunscreen (9.4% vs. 28%), staying in the shade (55.5% vs 65.5%), and using an umbrella (5.2% versus 12.1, %) compared to girls [23].

Other results of the study showed a significant relationship was also found between the students’ complexion and the attitude of protection against sunlight. The risk of most major skin cancers (including melanoma, basal cell carcinoma, and squamous cell carcinoma) is higher for individuals with a white complexion [24]. Therefore, people with white complexion show more protective behaviors against sunlight because they are more susceptible to sunlight [25].

The mean score of the sub-scale of behaviors leading to tanning was 77.76 ± 14.7 in all students, indicating their good level of control over tanning behaviors however some studies suggest that the youth find tanned skin tones more attractive than pale skin. Contrary to our study results, in the US, the incidence of skin tan was higher among women, younger people, and those with brightly colored skin and it seems tanning is still prevalent despite the well-publicized risks [15, 26]..

Our study showed that the mean score of the students’ protection behaviors was lower in those who thought they knew enough to protect themselves compared to those who believed they need to increase their protection level. These results indicate that there is no standard for people to compare their knowledge with an acceptable level of information, which makes them think this low level of knowledge and care is enough, resulting in no protection from sunlight. The results of the present study showed that protective behaviors like wearing clothes covering most of the body (long-sleeved dressing) and staying in the shade at peak hours of ultraviolet radiation were the most common behaviors. However, a sunshade was used less than other protective measures. Comparing the study results with other studies showed that sun protection behaviors vary in different cultures. In a study of college students tanning behaviors, attitudes, beliefs, and intentions, key motivators were appearance, emotion, health perceptions, and the influence of parents, peers, and the media [27].

Protective behaviors against sunlight of primary and secondary school students of northwestern Switzerland were investigated and it was found that the use of sunshades and suits was the least used ones [28]. In the assessment of knowledge, behavior and sun protection practices of vocational school students of health services in Istanbul was found that 91.5% of men and 95.5% of the women considered staying in the shade as the preferred method to protect themselves from the sun and it was the most common protective behavior among people [29].

Based on the results of the present study, students with less experience of sunburn had better sun protection behaviors scores in general. Moreover, most of the students (66.47%) never experienced sunburn. A large proportion of the Iranian population has a white complexion. These people are more sensitive to sunlight, which makes them adhere to basic protective behaviors. Sunburn is a significant risk factor for melanoma. Based on a study conducted in the United States, sunburn is seen in nearly 40% of the population each year, indicating that people do not adequately protect themselves from UV, which may cause melanoma. This study suggests that enhancing UV protective behaviors by multi-component community-wide programs and educational, environmental, and policy interventions based on evidence decreases skin damage that may develop into melanoma, and reduce health care expenditure [30].

However, based on the present study, knowledge, attitude, and behavior of students regarding sun protection were at an acceptable level. The percentage of correct answers to some questions was low, and few students were aware of some protective item such as vitamin D production in the skin through exposure to sunlight, the better impact of dark clothing versus bright clothing to protect from ultraviolet radiation, and the cumulative effect of ultraviolet exposure and the risk of tanning. Therefore, it is suggested to consider education based on local and regional guidelines in the field of sun protection.

## Conclusion

Since a high percentage of skin lesions are attributed to exposure to sunlight, public awareness and behaviors regarding sunlight protection are very important in prevention programs. In this study, knowledge, attitude, and protective behaviors of students aged 14–19 years were studied. The results showed moderate levels of knowledge and attitude a poor level of sun protection behavior. Base on the results of the study sex had a significant correlation with knowledge and behavior. Other results revealed a significant correlation between complexion color and the attitude towards sunlight protection. The mean score of protection behaviors was lower for those who believed they know enough to protect themselves than the students who believed they had to increase their protection level. Finally, analysis of the results showed behavior was largely influenced by cultural aspects and the implementation of the current guideline can be effective in reducing the health effects of sun exposure. Nevertheless, the option of environmental interventions, particularly attractive shade should be considered.

## Supplementary information

**Additional file 1.** Appendix A Sun protection questionnaire.

**Additional file 2.** STROBE Statement—Checklist of items that should be included in reports of cross-sectional studies.

## Data Availability

The data used and analyzed during the current study are available from the corresponding author upon reasonable request.

## Abbreviations

EPA: Environmental Protection Agency
IARC: International Agency for Research on Cancer
I-CVI: Item Content Validity Index
NTP: National Toxicology Program
S-CVI: Scale Content Validity Index
UVR: Ultraviolet Radiation.
UV: Ultraviolet
ACS: American Cancer Society
WHO: World Health Organization
