# Supplementary material for: Sun exposure and health safety practices of high school students in an urban population of Iran
Source: BMC Public Health. 2019 Dec 26;19:1736. doi: 10.1186/s12889-019-8100-7 (PMC6933744; doi:10.1186/s12889-019-8100-7)
Supplement: Supplementary file 1 — Additional file 1. Appendix A Sun protection questionnaire. [file 12889_2019_8100_MOESM1_ESM.docx]

**Sun exposure and health safety practices of high school students in an urban population of Iran**

Hosna Janjani^1^, Saharnaz Nedjat^2^, MasoudYunesian^1^, Simin Nasseri^1^, Fatemeh Haghighat Doost^1^, Ramin Nabizadeh^1^*

^1^Department of Environmental Health Engineering, School of Public Health, Tehran University of Medical Sciences, Tehran, Iran.

^2^ Department of Epidemiology and Biostatistics, School of Public Health, Tehran University of Medical Sciences, Tehran, Iran

**Appendix A**

**Sun protection questionnaire**

**Demographics information**

1. **Age……..**
2. **Sex………**
3. **Educational level**

First year of high school Second year of high school Third year in high school Fourth grade of high school

1. **Father educational level**

Illiterate Elementary Middle school High school and diploma Academic

1. **Mother educational level**

Illiterate Elementary Middle school High school and diploma Academic

1. **Skin color**

Pale white White Olive to brown Dark brown Black

1. **Knowledge**

Do you think the following statements are true or false?

| 1 | Small amounts of UV radiation are beneficial to health, and play an essential role in the production of vitamin D | True | False | I don’t know |
| --- | --- | --- | --- | --- |
| 2 | Long-term exposure to UV radiation from the sun has many health effects | True | False | I don’t know |
| 3 | Vitamin D is absorbed through the skin | True | False | I don’t know |
| 4 | Sunbathing damages our health | True | False | I don’t know |
| 5 | Ultraviolet radiation can play a contributory role in the development of various ocular disorders | True | False | I don’t know |
| 6 | Tanning in the sun is safe | True | False | I don’t know |
| 7 | The intensity of solar UV rays is the same throughout the day | True | False | I don’t know |
| 8 | Ultraviolet (UV) radiation from the sun can lead to skin cancer | True | False | I don’t know |
| 9 | If you do not feel the hot rays of the sun, You can't get sunburnt | True | False | I don’t know |
| 10 | If the skin is not protected from the sun, it will age and wrinkle faster | True | False | I don’t know |
| 11 | In a cloudy day there is no possibility of sunburn | True | False | I don’t know |
| 12 | Sunscreens protect the skin so you can stay in the sun for a long time if used | True | False | I don’t know |
| 13 | When sunbathing, we will not burn when give your skin a rest | True | False | I don’t know |
| 14 | Exposure to sunlight for a short period of time without protective measures is not a risk to the skin | True | False | I don’t know |
| 15 | Solar UV rays are not dangerous during the winter | True | False | I don’t know |
| 16 | Just elderly people who have been exposed to sunlight for many years get skin cancer | True | False | I don’t know |
| 17 | Skin cancer can be fatal | True | False | I don’t know |
| 18 | Tanning protects the body against the sun | True | False | I don’t know |
| 19 | Water, snow and sand surfaces such as beaches increase the risk of sunburn | True | False | I don’t know |
| 20 | Exposure to ultraviolet rays can cause cataracts | True | False | I don’t know |
| 21 | Exposure to sunlight from 11am to 4pm is very harmful to the skin | True | False | I don’t know |
| 22 | Skin cancer only occurs in white people | True | False | I don’t know |
| 23 | Natural or artificial tanning increases the risk of skin cancer. | True | False | I don’t know |
| 24 | Proper use of sunscreen is enough to protect yourself from the sun | True | False | I don’t know |
| 25 | Applying sunscreen once a day is effective against the sun's harmful rays | True | False | I don’t know |
| 26 | When using sunscreens, SPF (sun protection index) should be considered | True | False | I don’t know |
| 27 | You need to apply sunscreen 20 minutes before leaving the house | True | False | I don’t know |
| 28 | Applying sunscreen is the only way to protect you from the sun. | True | False | I don’t know |
| 29 | Staying in the shade or using the canopy does not provide complete protection against the sun's UV rays. | True | False | I don’t know |
| 30 | Light-colored garments provide better protection against solar UV rays | True | False | I don’t know |
| 31 | Wearing thick clothing cause less UV radiation penetrates the skin | True | False | I don’t know |
| 32 | Loose clothing provides better protection against sunlight. | True | False | I don’t know |
| 33 | Wet clothes increase the transmission of UV rays | True | False | I don’t know |
| 34 | Sunscreen should have the SPF of at least 15 | True | False | I don’t know |
| 35 | If you use a hat you do not need to use sunscreen | True | False | I don’t know |
| 36 | Sunscreens should be reapplied approximately every two hours when outdoors, even on cloudy days | True | False | I don’t know |
| 37 | After swimming there is no need to reuse the sunscreen. | True | False | I don’t know |
| 38 | Apply sunscreen only to your face | True | False | I don’t know |
| 39 | To protect against the sun, in addition to sunscreen, wearing hats, glasses and appropriate clothing is also essential. | True | False | I don’t know |
| 40 | Gradually tanning is safe until it does not burn | True | False | I don’t know |

1. **Attitude**

How do you think the effect of the following behaviors is to protect you from the sun?

| Wearing broad-brimmed hats | Very high | High | Medium | Low | No effect |
| --- | --- | --- | --- | --- | --- |
| Apply sunscreen with SPF of at least 15 | Very high | High | Medium | Low | No effect |
| Stay in the shade away from the sun | Very high | High | Medium | Low | No effect |
| Wear appropriate clothing that protects the skin (long sleeves, etc.) | Very high | High | Medium | Low | No effect |
| Use proper sunglasses. (With UV absorbent coating that covers all eyes and around it | Very high | High | Medium | Low | No effect |

To answer the following questions, imagine being outdoors on a warm sunny day. How often do you do listed activities?

| 1 | Exposure to sunlight for tanning | Never | rarely | sometimes | often | always |
| --- | --- | --- | --- | --- | --- | --- |
| 2 | Using of cream, lotion or low SPF oil to tanning | Never | rarely | sometimes | often | always |
| 3 | No timely use of sunscreen against sun rays to tanning | Never | rarely | sometimes | often | always |
| 4 | Wear short dress to expose the skin to sunlight for tanning | Never | rarely | sometimes | often | always |
| 5 | Activities such as sports, swimming and hiking to tanning | Never | rarely | sometimes | often | always |

**c) Practice**

1) How often do you go to your doctor to check your skin status?

- Once a month - Every 1-12 months - Every 1-2 years - Less than once every 2 years - Never

- Only when I have problem

2) How many times in the past 12 months have you experienced a painful sunburn or redness of the skin lasting 1 day or more?

- Never - Once every 6 months - Once every 2-3 months - Every month - every week

3) Choose one of the following sentences that is the best option for describing your performance outside the home (just select one).

1. I know I need to protect myself from the sun, and I always do
2. I like tanning but I use sunscreen because I don't want to get premature skin aging and other side effects.
3. I like to be tanned so I don't use sun protection
4. I try to use protective methods, but I often forget
5. My skin is dark and I get very little or rarely sunburn so I don't need to protect myself from the sun
6. I don't usually use sunscreen because it takes my time and makes my skin feel bad.

|  | How much of the following activities do you do? (Select only one item per question) | | | | | |
| --- | --- | --- | --- | --- | --- | --- |
| 1 | Use protective glasses against UV light | Never | rarely | sometimes | often | always |
| 2 | Wear a broad-brimmed hat to protect yourself from the sun | Never | rarely | sometimes | often | always |
| 3 | Apply sunscreen before going out | Never | rarely | sometimes | often | always |
| 4 | Use sunscreen for all parts of the body that are exposed to the sun | Never | rarely | sometimes | often | always |
| 5 | reapply sunscreen every 2 hours when you're out | Never | rarely | sometimes | often | always |
| 6 | reapply sunscreen after swimming | Never | rarely | sometimes | often | always |
| 7 | Apply sunscreen when practice in the snow (skiing, …) | Never | rarely | sometimes | often | always |
| 8 | Staying home and avoiding sun exposure between 11am and 4pm | Never | rarely | sometimes | often | always |
| 9 | Stay in the shade when you are out between 11am and 4pm | Never | rarely | sometimes | often | always |
| 10 | Using canopies (like umbrellas) when you are out between 11am and 4pm | Never | rarely | sometimes | often | always |
| 11 | Wearing appropriate clothing that protects the skin (long sleeves and ... | Never | rarely | sometimes | often | always |
| 12 | Use of sun protection methods on cloudy days | Never | rarely | sometimes | often | always |

•If you wear a hat, does it have a width of 7.5 cm? -Yes -No -I don't wear a hat

•If you wear a hat, does it have a cover for the back and neck? -Yes -No -I don't wear a hat

What do you think about protecting yourself from the sun right now?

- At the moment I know that I have enough protection

-I can better protect myself
